# RNA splicing process analysis for identifying antisense oligonucleotide inhibitors with padlock probe-based isothermal amplification†
†Electronic supplementary information (ESI) available: Additional experimental materials, methods, DNA sequences and supplementary figures and tables. See DOI: 10.1039/c7sc01336a



**DOI:** 10.1039/c7sc01336a

**Published:** 2017-06-13

**Authors:** Xiaojun Ren, Ruijie Deng, Lida Wang, Kaixiang Zhang, Jinghong Li

**Affiliations:** a School of Chemistry and Chemical Engineering , Beijing Institute of Technology , Beijing 100081 , China; b Department of Chemistry , Key Laboratory of Bioorganic Phosphorus Chemistry & Chemical Biology , Tsinghua University , Beijing 100084 , China . Email: jhli@mail.tsinghua.edu.cn

## Abstract

RNA splicing, which mainly involves two transesterification steps, is a fundamental process of gene expression and its abnormal regulation contributes to serious genetic diseases. Antisense oligonucleotides (ASOs) are genetic control tools that can be used to specifically control genes through alteration of the RNA splicing pathway. Despite intensive research, how ASOs or various other factors influence the multiple processes of RNA splicing still remains obscure. This is largely due to an inability to analyze the splicing efficiency of each step in the RNA splicing process with high sensitivity. We addressed this limitation by introducing a padlock probe-based isothermal amplification assay to achieve quantification of the specific products in different splicing steps. With this amplified assay, the roles that ASOs play in RNA splicing inhibition in the first and second steps could be distinguished. We identified that 5′-ASO could block RNA splicing by inhibiting the first step, while 3′-ASO could block RNA splicing by inhibiting the second step. This method provides a versatile tool for assisting efficient ASO design and discovering new splicing modulators and therapeutic drugs.

## Abstract 2

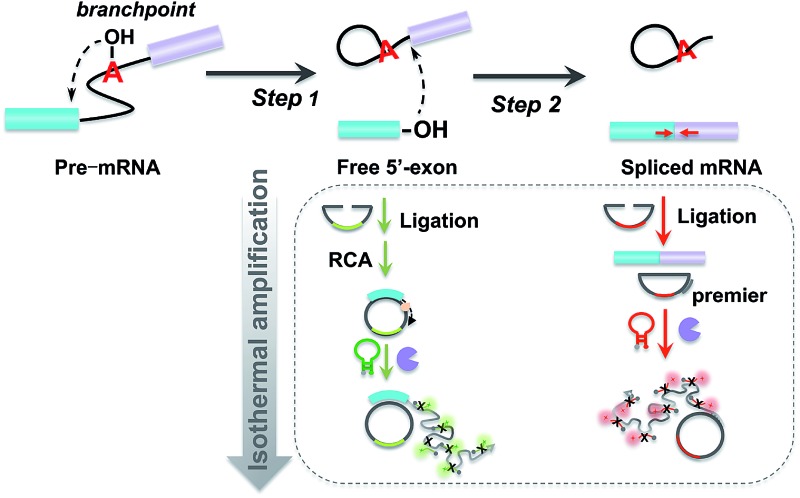
We report a highly sensitive method for quantifying the splicing products in different steps, enabling us to analyze the splicing process and identify ASO inhibitors.

## Introduction

RNA splicing, a fundamental process for gene expression comprising two sequential transesterification reactions,^1^ plays an important role in proteome complexity and gene expression regulation.^2,3^ This process must proceed under strict regulatory control.^4–7^ Aberrant splicing has been implicated in numerous diseases and conditions such as cancer and neurodegeneration.^8–11^ Splicing is a favourable intervention point for disease therapeutics since it is an early step in gene expression and does not alter the genome.^12^ Antisense oligonucleotides (ASOs) function by hybridizing to pre-mRNA in a sequence-specific manner and sterically block access of splicing factors to the target site such that the splicing pathways are altered.^13,14^ Given the advantages such as high substrate specificity, low toxicity, enduring effects and ease of delivery, ASOs are commonly used tools to correct or alter RNA expression for therapeutic benefits and have been applied to genetic disorders in several human clinical trials.^15–17^

Despite intensive research, how ASOs or various other factors influence the multiple processes of RNA splicing remains obscure. RNA splicing takes place in multiple sequential transesterification steps, which will generate RNA products with the same sequence components, such as free 5′-exon and spliced mRNA, or with complex structures, such as lariat 3′-exon.^18–21^ Due to the lack of a sensitive and easy method that could be used to simultaneously distinguish and quantify the intermediate and final splicing products, the efficiency of each step in the RNA splicing reaction is unable to be analysed. Gel electrophoresis is the golden method for *in vitro* RNA splicing analysis.^22^ However, it has relatively low throughput and needs radioisotopic labels to achieve high sensitivity, which makes it time-consuming and laborious. Besides gel electrophoresis, methods such as reverse-transcription PCR (RT-PCR)^23,24^ and microarrays^25^ for RNA splicing analysis cannot be adapted to analyze the multiple-step process, as they hardly discriminate between the intermediate and final products, which share the same sequence. Most recently, several techniques have been applied to RNA splicing analysis, such as ligation-dependent PCR,^26^ single-molecule fluorescence resonance energy transfer (smFRET)^27^ and plasmonic nanoparticles.^28^ Still, they are all not amenable to detect the intermediate splicing products and thus cannot be used for quantitative evaluation of the two-step splicing efficiency. The identification of ASOs or various other factors that play a role in specific steps^29^ of splicing necessitates the development of methods for dissecting multiple RNA splicing processes.

Herein, to solve this issue we develop a padlock probe-based isothermal amplification assay to precisely detect the specific products of each step, free 5′-exon and spliced mRNA, enabling us to analyze the splicing process and study its regulation. The padlock probe-based amplification reaction has previously been reported by our group^30^ and other researchers,^31,32^ and exhibits excellent sensitivity and specificity for RNA detection. The padlock probe is introduced as a rolling circle amplification template,^33,34^ enabling the specific identification of multiple target RNA sequences, including the intermediate and final products. Moreover, the subsequent nicking endonuclease-based enzymatic recycling cleavage process^35,36^ with high amplification ability confers highly sensitive detection of the RNA splicing products. With this amplified assay, the roles of ASOs in inhibiting RNA splicing at the first and second steps can be successfully distinguished. The effects of five ASOs binding different sites on RNA splicing have been examined and the ASOs are shown to block the splicing process at various stages. Notably, we identify that 5′-ASO can block RNA splicing by inhibiting the first step, while 3′-ASO can block RNA splicing by inhibiting the second step. Thus, this approach not only helps us to further understand the RNA splicing process but also shows huge potential in the discovery of splicing modulators and therapeutic drugs.

## Results and discussion

### Principle of padlock probe-based isothermal amplification assay for analyzing RNA splicing

**Scheme 1:**
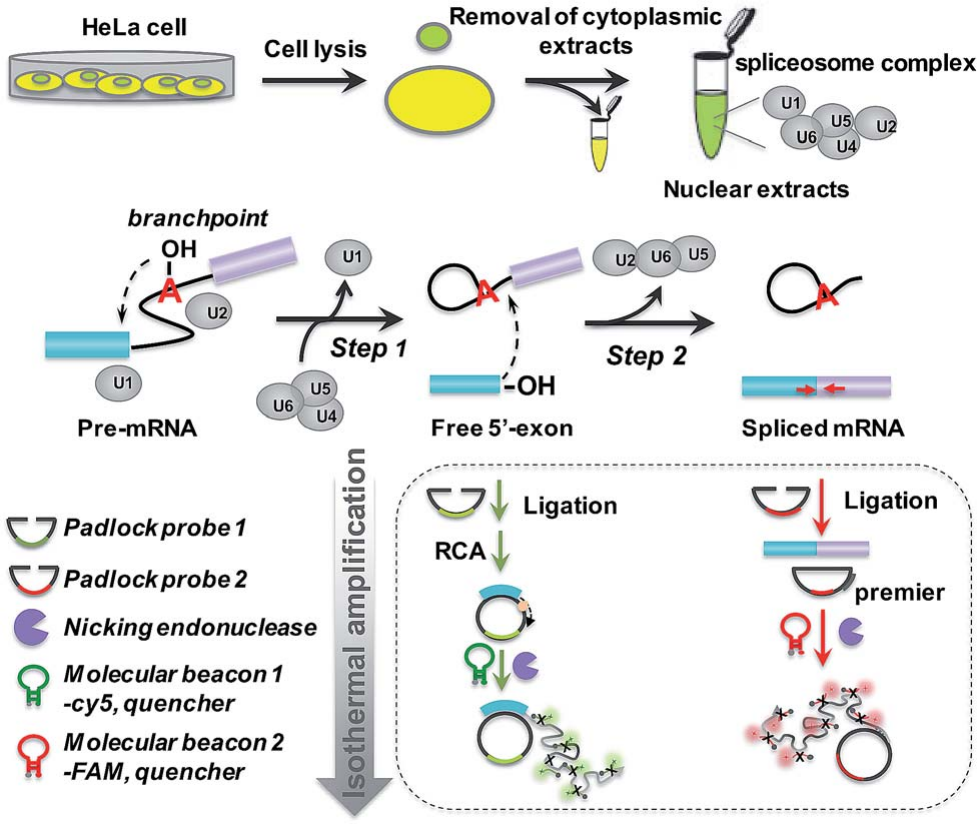
Schematic diagram of the padlock probe-based isothermal amplification assay for analyzing RNA splicing. (1) Nuclear extracts (NE) containing spliceosome complex are separated from HeLa cells for an *in vitro* RNA splicing assay. U1, U2, U4, U5 and U6 are major small nuclear ribonucleoproteins (snRNPs) that form the catalytic core of the spliceosome. (2) Under the catalysis of the spliceosome complex, the free 5′-exon is generated in the first step. In the second step, the free 5′-exon attacks the 3′ splice site, yielding ligated exons (spliced mRNA). (3) Padlock probes 1 (green) and 2 (red) are designed to specifically hybridize to the free 5′-exon and spliced mRNA, respectively. (4) Following these two recognition processes of the padlock probes, the amplified assay is realized through ligation, rolling circle amplification and nicking endonuclease-assisted fluorescence signal amplification.

The principle of the padlock probe-based isothermal amplification assay for RNA splicing analysis is illustrated in Scheme 1. Initially, nuclear extracts (NE) containing a spliceosome complex are acquired from HeLa cells to perform RNA splicing *in vitro*.^37^ Under the catalysis of the spliceosome complex, the first transesterification step will produce the free 5′-exon. Then the two exons are ligated together to form a mature mRNA in the second step. For the detection of the free 5′-exon, the 3′ end sequence in the free 5′-exon is used as a template to cyclize padlock probe 1 and subsequently as a primer to trigger the RCA reaction. For the detection of spliced mRNA, the newly formed junction sequence in the mature spliced mRNA is specifically targeted by padlock probe 2 and then RCA is initiated with the help of an additional DNA primer. The RCA process is followed by an enzymatic recycling cleavage reaction to further improve the sensitivity of the assay. A molecular beacon (MB) signal reporter probe, which is designed to be complementary to the rolling circle products (RCPs), contains a recognition site for nicking endonuclease. The nicking endonuclease can recognize the specific double-stranded sequence between MB and RCP and cleave MB, resulting in the complete disconnection of the fluorophore from the quencher, and then the fluorescence signal is turned on. MBs 1 and 2 for recognition of the different RCPs, the target free 5′-exon and spliced mRNA are modified with Cy5 and 5′-FAM, respectively. These two fluorescence signals at 660 nm and 520 nm specifically reflect the amount of free 5′-exon and spliced mRNA. By recording the fluorescence intensity, we can quantify the different RNA splicing products and evaluate the efficiency of the first-step and second-step RNA splicing simultaneously.

### Amplified assay for detection of the two-step RNA splicing products

**Fig. 1:**
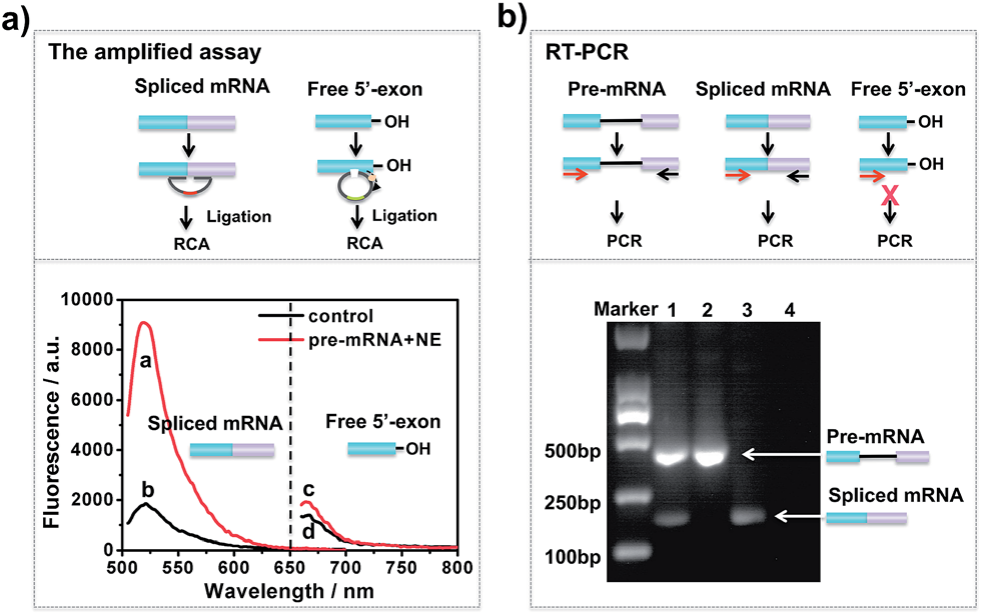
Comparison of the padlock probe-based isothermal amplification assay with the RT-PCR assay for detection of the two-step RNA splicing products. The major difference is that the amplified assay can be used for simultaneous detection of the intermediate and final products, while RT-PCR could not detect the intermediate products. (a) Fluorescence responses of the isothermal amplified assay under different conditions: *in vitro* splicing assays were conducted in the presence of NE (red curve) or absence of NE (black curve). The splicing products were analysed by the padlock probe-based isothermal amplified assay. (b) Gel electrophoresis of the RT-PCR products for *in vitro* RNA splicing: 40 pM CDC pre-mRNA was incubated in the presence of NE (lane 1) or absence of NE (lane 2) for 1 h at 30 °C under the conditions of *in vitro* RNA splicing; lane 3, spliced mRNA; lane 4, NE.

To investigate whether the developed approach could effectively evaluate the two-step splicing efficiency, a shortened version of chicken δ-crystallin (CDC) pre-mRNA^38^ containing two exons (exons 14 and 15) and an intron was used as a splicing substrate for the *in vitro* splicing reaction. As shown in Fig. 1a, in the presence of NE, a strong fluorescence signal was observed for spliced mRNA (curve a), while free 5′-exon showed a low fluorescence signal (curve c). In the control reaction without NE, the fluorescence intensities of both spliced mRNA and free 5′-exon were low and unchanged (curves b and d). This is because when the pre-mRNA underwent both transesterification steps under the catalysis of NE, the amount of spliced mRNA generated was far more than that of free 5′-exon. In the absence of NE, the splicing reaction could not occur and neither free 5′-exon nor spliced mRNA was generated to trigger the amplification reaction. Thus, the established approach is capable of detecting free 5′-exon and the spliced mRNA generated by the *in vitro* RNA splicing reaction.

RT-PCR analysis was carried out in parallel for comparison. The results of the reactions were analysed by 1% agarose gel. As shown in Fig. 1b, when CDC pre-mRNA was spliced, there were two bands indicating the unspliced pre-mRNA and spliced mRNA, respectively (lane 1). However, without pre-mRNA or NE, the splicing reaction couldn’t take place and thus no band for spliced mRNA was shown in the gel (lanes 2 and 4). RT-PCR could detect the final spliced products, however, it can hardly detect the intermediate product. The developed amplified assay could probe both the intermediates and final products simultaneously, thus enabling analysis the each step efficiency in RNA splicing.

### Sensitivity and specificity of the amplified assay

**Fig. 2:**
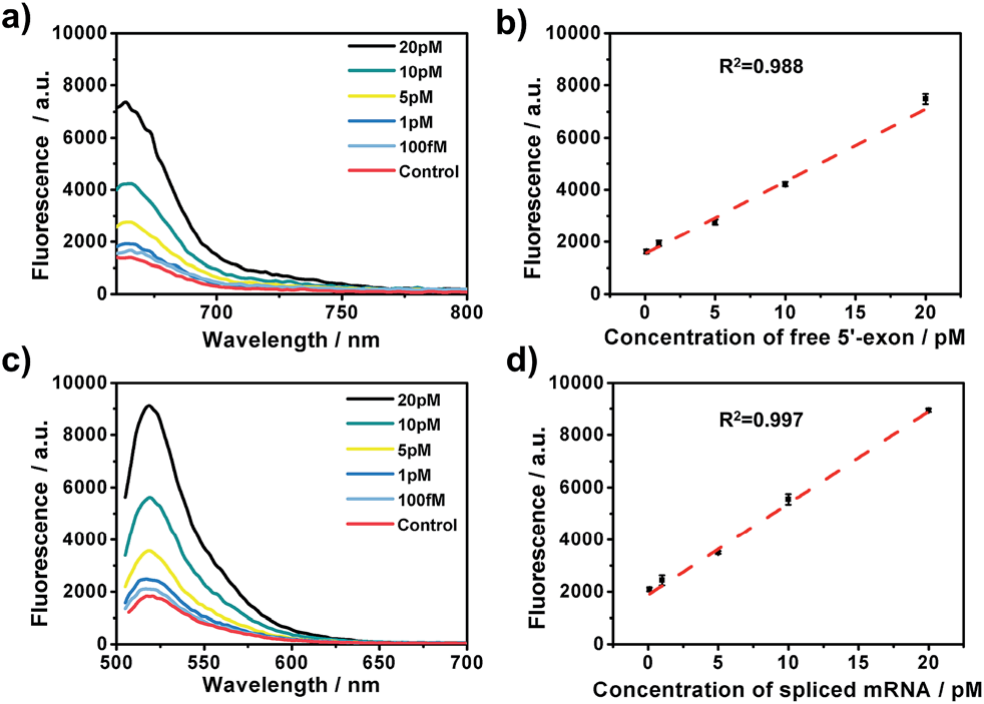
The amplified assay for quantification of the two-step RNA splicing products. (a and c) Fluorescence emission spectra in the presence of different concentrations of free 5′-exon and spliced mRNA (from top to bottom: 20 pM, 10 pM, 5 pM, 1 pM, 100 fM, and control). (b) Linear relationship between the fluorescence intensities at 660 nm and concentrations of free 5′-exon in (a). (d) Linear relationship between the fluorescence intensities at 520 nm and concentrations of spliced mRNA in (c). Error bars are based on triplicate experiments. The concentrations of padlock probe, Bst polymerase and Nb.Mva1269I were 10 nM, 5 U and 10 U, respectively.

After the optimization of the concentration and incubation time of Bst DNA polymerase and the nicking endonuclease (Fig. S1†), the sensitivity of the amplified assay was tested. A series of concentrations of free 5′-exon and spliced mRNA were detected under the optimum conditions. As shown in Fig. 2, free 5′-exon and spliced mRNA could be quantitatively detected in a linear range of 100 fM to 20 pM. The fluorescence intensities at 660 nm corresponded linearly to the concentrations of free 5′-exon, with the correlation equation FI = 1449.68 + 286.18[free 5′-exon] (*R* ^2^ = 0.988). The fluorescence intensities at 520 nM corresponded linearly to the concentrations of spliced mRNA, with the correlation equation FI = 1881.98 + 350.49[spliced mRNA] (*R* ^2^ = 0.997). The detection limits (at 3 times the standard deviation of the background) of free 5′-exon and spliced mRNA were estimated to be 81 fM and 29 fM, respectively. Thus this is a highly sensitive RNA detection assay which can be used for quantification of the products in the RNA splicing reaction.

**Fig. 3:**
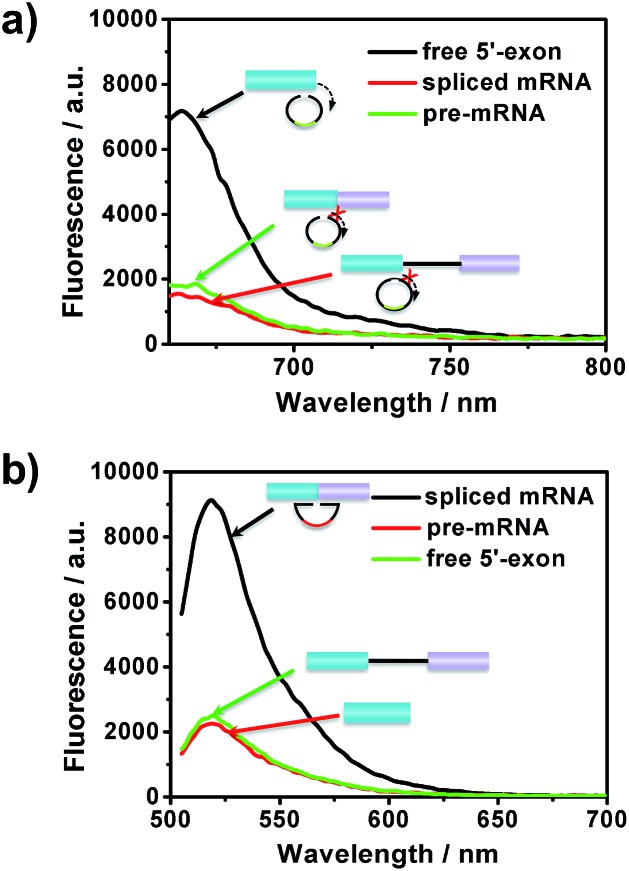
The specificity of the amplified assay for the detection of different RNA splicing products: (a) free 5′-exon and (b) spliced mRNA. Padlock probes 1 and 2 were incubated with 20 pM free 5′-exon, CDC pre-mRNA and spliced mRNA, respectively.

The specificity of the assay is key for distinguishing different RNA products in the splicing reaction. To investigate the specificity of the described approach, pre-mRNA, free 5′-exon and spliced mRNA were tested by padlock probes 1 and 2, respectively. Padlock probe 1 was designed to be complementary to the 3′ end sequence of the free 5′-exon. After the padlock-probe circularization reaction, the free 5′-exon was used as a primer to be extended to hundreds of tandem repeats by RCA. The pre-mRNA and spliced mRNA can hardly be directly recognized by padlock probe 1 and trigger the RCA, because padlock probe 1 hybridized to the RNA sequence at a distance far from the 3′-end of the pre-mRNA or spliced mRNA. In Fig. 3a, a more obvious signal enhancement in the fluorescence intensity was observed upon addition of free 5′-exon than that with pre-mRNA and spliced mRNA, indicating that the assay could discriminate free 5′-exon from pre-mRNA and spliced mRNA. Padlock probe 2 was designed to target the junction sequence in spliced mRNA. As shown in Fig. 3b, the fluorescence signal in the presence of spliced mRNA was significantly higher than that with pre-mRNA or free 5′-exon, indicating that padlock probe 2 could specifically recognize the spliced mRNA. These two results indicate that the proposed approach could offer high specificity to distinguish different RNA splicing products.

### Effect of binding sites of ASOs on RNA splicing efficiency

**Fig. 4:**
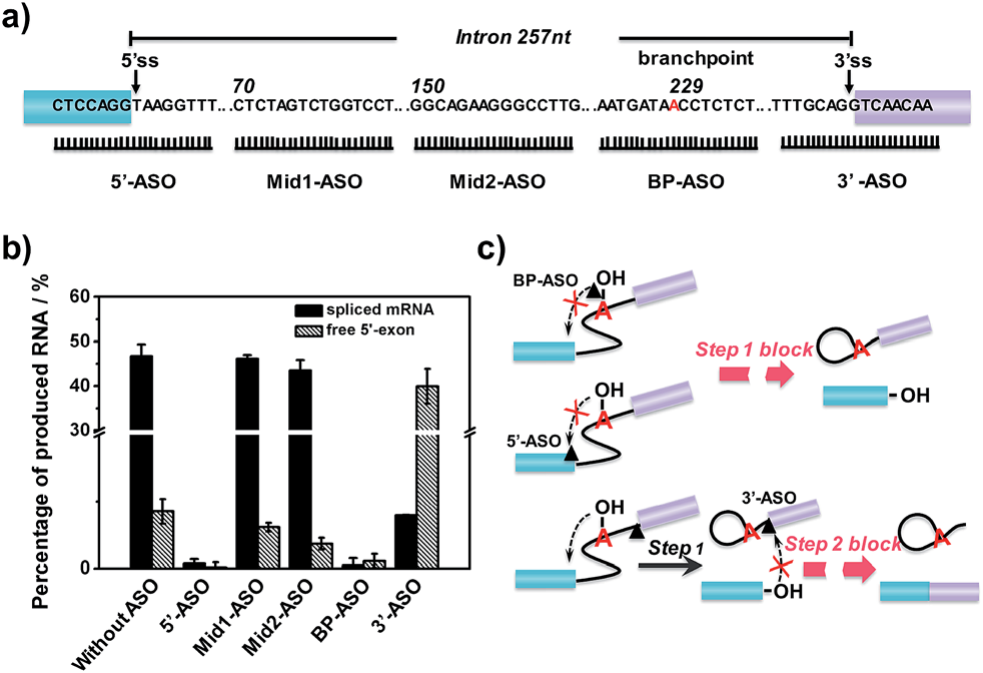
Effect of ASO binding sites on first- and second-step RNA splicing efficiency. (a) Diagrammatic representation of ASO targeting sequences (5′-ASO, Mid1-ASO, Mid2-ASO, BP-ASO and 3′-ASO). Numbering starts from the first position of intron 14. The 5′ss and 3′ss are indicated by vertical arrows. The branchpoint is indicated in red. (b) Fluorescence intensity of free 5′-exon and spliced mRNA produced in the splicing reaction by adding different ASOs. (c) Schematic diagram of the splicing block caused by ASOs binding different sites.

After verification and optimization, we explored the potential of the approach to study ASOs’ inhibitory effects in RNA splicing. Some ASOs targeting the splicing process have been identified and evaluated as drug candidates.^39–41^ RNA splicing activity is modulated by ASOs, and ASOs with different RNA targeting sites have been demonstrated to exhibit different effects on the splicing events.^42^ We designed five ASOs targeting different RNA sites, as shown in Fig. 4a. Three ASOs, binding to the 5′ splice site (5′ss), branchpoint sequence (BPS) and 3′ splice site (3′ss), were designed as these sites are core recognition sites in the splicing reaction. The other two ASOs (Mid1-ASO and Mid2-ASO), binding to sites that are far from these core sites, were designed for comparison. Besides, we used 15-mer 2′-*O*-methoxyethyl ribose (MOE)-modified phosphodiester ASOs, as ASOs with MOE modification show increased nuclease resistance and enhanced affinity for hybridization to complementary RNA, thus suppressing degradation of the target mRNA by RNase H.^43^

As shown in Fig. 4b, neither the amount of spliced mRNA nor free 5′-exon was detected when using 5′-ASO (binding to the 5′ splice site) and BP-ASO (binding to the branchpoint). This suggested that the two ASOs could strongly inhibit the splicing process at an early step. When the splicing reaction was conducted with Mid1-ASO (complementary to 70 to 84 nt) and Mid2-ASO (complementary to 151 to 165 nt), however, the amount of produced RNA products was roughly similar to that without ASOs. Thus, ASOs binding to sites that are far from these core sites produced a negligible inhibitory effect on the RNA splicing process. Remarkably, the addition of 3′-ASO, which targets the 3′ splice site, led to a pronounced decrease of spliced mRNA but a dramatic increase of the free 5′-exon, indicating that the pre-mRNA underwent the first step efficiently, whereas the second step was blocked. Given these results, all ASOs binding to core sites, such as the 5′ splice site, branchpoint or 3′ splice site, exhibited significant inhibitory effects on RNA splicing. However, as the RNA splicing was a multiple-step process, they were involved in different stages: ASO binding to the 5′ splice site or branchpoint mainly inhibited the first transesterification step, while ASO binding to the 3′ splice site inhibited the second transesterification step. The different inhibitory effects of the ASOs resulted from their different mechanisms for the modulation of RNA splicing. 5′-ASO and BP-ASO created a steric block to the binding of splicing factors to the 5′ splice site and branchpoint during the assembly in the first step, thus inhibiting attack of the 5′ splice site by branch adenine. 3′-ASO masking the 3′ splice site prevented the identification of the 3′ splice site and thus led to inhibition of the attack of the 3′ splice site by the free 5′-exon in the second step (Fig. 4c).

Furthermore, with this assay, the effect of branchpoint proximity (the distance between the branchpoint and the 3′ splice site) on the RNA splicing efficiency was investigated. We have identified that the second step of splicing could be completely blocked when the distance was shortened by 9 nt (Fig. S2†). The developed assay could not only be used for the identification of ASOs but could also be amenable to studying various splicing regulators in the RNA splicing process.

### Discriminating between different ASOs’ inhibitory effects for RNA splicing

**Fig. 5:**
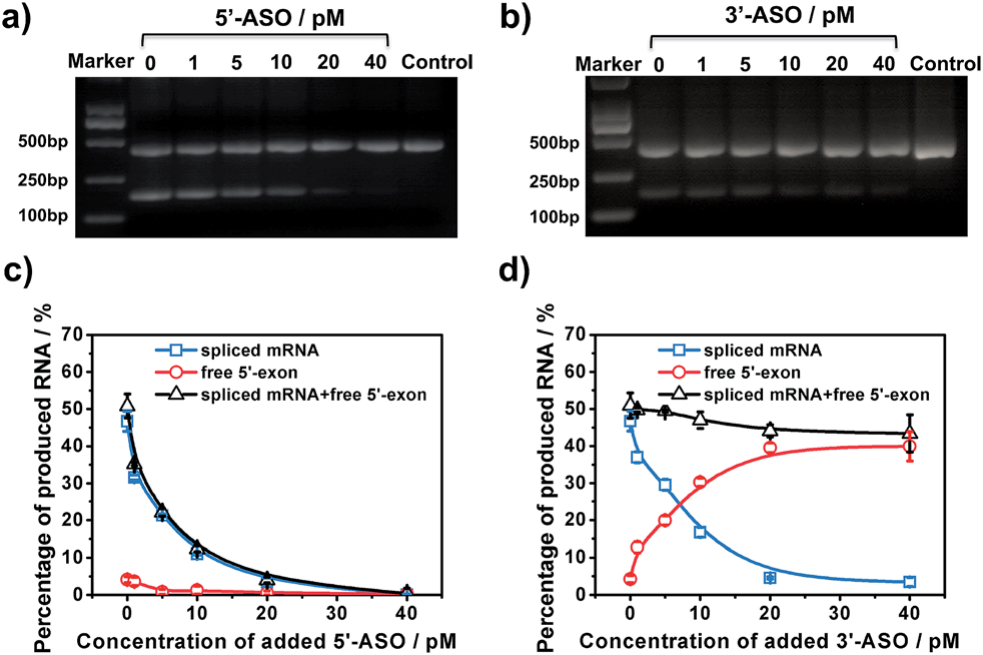
Analysis of 5′-ASO and 3′-ASO regulated RNA splicing processes. (a and b) Gel electrophoresis of the RT-PCR assay: *in vitro* RNA splicing was performed in NE in the presence of increasing amounts of 5′-ASO or 3′-ASO (0 pM, 1 pM, 5 pM, 10 pM, 20 pM and 40 pM). (c and d) Fluorescence responses in different concentrations of 5′-ASO or 3′-ASO (0 pM, 1 pM, 5 pM, 10 pM, 20 pM and 40 pM).

To further examine the different inhibitory effects of 5′-ASO and 3′-ASO on splicing, we performed RNA splicing assays by adding a series of ASO concentrations (0, 1, 5, 10, 20 and 40 pM). ASOs were mixed with pre-mRNA prior to the *in vitro* splicing reaction. RT-PCR experiments were performed in parallel to compare the two methods. As shown in Fig. 5a and b, along with the increase of the concentration of the ASO, the amount of the final product, spliced mRNA, decreased, and almost no band was found in 40 pM. This indicated that RNA splicing would be completely blocked by 40 pM ASO, and these two ASOs showed nearly the same inhibitory effect in the results of the RT-PCR experiments. However, with our amplified assay, the two ASOs showed different results. As shown in Fig. 5c, for 5′-ASO, with the increase of the ASO concentration, the amount of spliced mRNA decreased, while the amount of free 5′-exon remained at a low level and showed no obvious change, indicating that 5′-ASO prevented the production of both the splice intermediates and spliced mRNA. This revealed that 5′-ASO blocked splicing at an early step, thus almost no free 5′-exon was accumulated. However, for 3′-ASO, as shown in Fig. 5d, as the ASO concentration increased from 0 pM to 40 pM, the amount of spliced mRNA decreased, while the free 5′-exon showed a dramatic increase, indicating that 3′-ASO blocked the second-step splicing and thus caused free 5′-exon to accumulate. Furthermore, the total amount of free 5′-exon and spliced mRNA showed no significant change along with the increase in 3′-ASO concentration, thus revealing that 3′-ASO exhibited a modest inhibitory effect in the first step. When the 5′-ASO or 3′-ASO concentration reached 20 pM, the inhibitory efficiencies were calculated to be 92.61% and 90.27% (Table S2†), further indicating that both 5′-ASO and 3′-ASO showed a pronounced inhibitory effect. Compared with the RT-PCR analysis, our developed assay could distinguish the first- and second-step roles of ASOs in RNA splicing inhibition, which further verified that our approach could be applied for the quantitative analysis of RNA splicing regulation at different stages. Besides, ASOs are proposed as therapeutic agents to alter or correct gene function in diseases. The amplified assay provides a versatile tool for assisting efficient ASO design and ASO identification for drug discovery efforts.

## Conclusions

In summary, we developed a padlock probe-based isothermal amplification assay for precisely detecting the splicing products of different steps, enabling us to dissect the splicing process and study ASO regulation. By taking advantage of rolling circle amplification and enzymatic recycling cleavage, highly sensitive and specific recognition of free 5′-exon and spliced mRNA was achieved. With this amplified assay, the first- and second-step roles of ASOs in RNA splicing inhibition could be distinguished. The effects of five ASOs binding different sites on RNA splicing were examined and they were shown to block the splicing process at various stages. Notably, we identified that 5′-ASO could block RNA splicing by inhibiting the first step, while 3′-ASO could block RNA splicing by inhibiting the second step. This method enables the simultaneous analysis of multiple-step RNA splicing, which not only provides a useful tool for assisting efficient ASO design, but also shows potential in the investigation of the RNA splicing mechanism and identification of regulation factors as well as applications in drug discovery.

## Experimental section

### Materials and reagents

T4 DNA ligase (5 Weiss U μL^–1^), dNTP mix (10 mM), Nb.Bsml (10 000 U mL^–1^) and RiboLock RNase inhibitor (40 U μL^–1^) were purchased from Thermo Scientific. HiScribeTM T7 Quick High Yield RNA Synthesis Kit, Bst DNA polymerase 2.0 and AMV reverse transcriptase (1000 U mL^–1^) were purchased from NEB (New England Biolabs, Ipswich, MA, USA). PrimeSTAR®HS (Premix) used for PCR was obtained from TaKaRa Biotechnology Co., Ltd. (Dalian, China). All oligonucleotides were purchased from Shanghai Sangon Biological Engineering Technology and Services Co., Ltd. (Shanghai, China). All the padlock probes were modified with a 5′-phosphate group. The two molecular beacons were modified with Cy5 and 5′-FAM (5-carboxyfluorescein), both purified by HPLC. The RNA sequences were purchased from TaKaRa Biotech Company (Dalian, China).

### *In vitro* transcription of capped pre-mRNA and mRNA

Chicken δ-crystallin (CDC) mRNA was transcribed *in vitro* by a HiScribeTM T7 Quick High Yield RNA Synthesis Kit (purchased from NEB). The pre-mRNA was capped in the transcription reaction by incorporating m7GpppG (the ratio of m7GpppG to GTP was 4 : 1). Briefly, the *in vitro* transcription reaction was performed in 20 μL volumes at 37 °C for 1 h with 1 μg template DNA, 2 μL NTP buffer mix (5 mM of each NTP) and 2 μL T7 RNA polymerase mix. Then to remove the template DNA, 2 μL of DNase I (RNase-free, U mL^–1^) was added and the mixture was incubated at 37 °C for 20 min. The transcribed pre-mRNA was purified by Trizol and redissolved in RNase-free water. The purified pre-mRNA was qualified by Nanodrop 2000 and then stored at –80 °C for future use.

### Preparation of splicing extracts and the *in vitro* splicing reaction

Whole-cell splicing extracts from HeLa cells were prepared by a Nuclear/Cytosol Fractionation Kit (from BioVision) according to the manufacturer’s instructions. The *in vitro* splicing assay was performed essentially as described previously.^37^ Briefly, 50 μL of a reaction mixture containing 40 pM of capped pre-mRNA, 50 μg of HeLa cell nuclear extract, 1× SP buffer (20 mM creatine phosphate, 2.5 mM ATP, 20 mM MgCl_2_), 1 U μL^–1^ RNase inhibitor (Thermo Fisher) and 13% PVA (added last) was incubated at 30 °C for 60 min.

### *In vitro* RNA splicing activity analysis by RT (reverse transcription)-PCR

In the PCR analysis, the forward primer was 5′-AGGAAGCTGTCCTTGATGTT, and the reverse primer was 5′-CTACGTCTCGGTCTAGTCATC. The reverse primer was also used as a primer for reverse transcription. The splicing reaction products were first purified by Trizol and phenol. Then the reverse transcription was performed in a 20 μL reaction mixture containing 1× AMV reverse transcriptase buffer [50 mM Tris–HCl (pH 8.3), 75 mM KOAc, 8 mM Mg(OAc)_2_, 10 mM DTT, 1 μL reverse primer, dNTP (10 mM) and 10 U AMV reverse transcriptase]. The reaction buffer was incubated for 30 min at 42 °C and heated to 80 °C for 10 min. Next, 2 μL of the product was mixed with 10 μL PrimeSTAR®HS, 1 μL forward primer and 1 μL reverse primer and diluted to a final volume of 20 μL for PCR. The PCR product was analysed by 2% agarose gel electrophoresis.

### Ligation reaction and rolling circle amplification

The ligation reaction was conducted at 25 °C for 1 h in a 20 μL reaction mixture containing 1× T4 ligation buffer [40 mM Tris–HCl, 10 mM MgCl_2_, 10 mM DTT, 0.5 mM ATP (pH 7.8 at 25 °C)], 5 μL of splicing reaction products, 2 μL of the padlock probe (1 μM) and 5 U of T4 ligase. Then 0.5 μL Bst DNA polymerase, 5 μL dNTPs and 1× isothermal amplification buffer [20 mM Tris–HCl, 10 mM (NH_4_)_2_SO_4_, 50 mM KCl, 2 mM MgSO_4_, 0.1% Tween 20 (pH 8.8 @ 25 °C)] were added to the above reaction mixture. After 1 h incubation at 65 °C, the reaction was stopped by heating to 80 °C for 10 min.

### Fluorescence assay procedures

To perform the fluorescence assay, the prepared reaction mixture was mixed with 200 nM MB and 1 μL Nb.Bsml and diluted to a final volume of 50 μL. After 30 min incubation at 37 °C, 30 μL of the solution was transferred to a cuvette and the fluorescence spectra were measured on a Cary Eclipse (Varian). The excitation wavelength was 488 nm for FAM and 646 nm for Cy5, and the emission wavelengths were 520 and 660 nm, respectively.
