# Supplementary material for: RNA splicing process analysis for identifying antisense oligonucleotide inhibitors with padlock probe-based isothermal amplification
Source: Chem Sci. 2017 Jun 13;8(8):5692–8. doi: 10.1039/c7sc01336a (PMC5621167; doi:10.1039/c7sc01336a)
Supplement: Supplementary file 1 [file SC-008-C7SC01336A-s001.pdf]

Electronic Supplementary Material (ESI) for Chemical Science.
This journal is © The Royal Society of Chemistry 2017

Supporting Information

RNA splicing process analysis for identifying antisense oligonucleotide inhibitors with padlock probe-based isothermal amplification

*Xiaojun Ren[a,b], Ruijie Deng[b], Lida Wang[b], Kaixiang Zhang[b], Jinghong Li[b]**

a. School of Chemistry and Chemical Engineering, Beijing Institute of Technology, Beijing 100081, China

b. Department of Chemistry, Key Laboratory of Bioorganic Phosphorus Chemistry & Chemical Biology, Tsinghua University, Beijing 100084, China

* To whom correspondence should be addressed. Email: jhli@mail.tsinghua.edu.cn

Table of contents

1. Table S1. Oligonucleotide sequences S2
2. Table S2. Inhibitory efficiency by different concentrations of ASO S3
3. Figure S1. Enzyme concentration and incubation time optimization S4
4. Effect of the distance between the branchpoint and the 3' splice site on RNA splicing efficiency S5
5. Figure S2. Effect on RNA splicing efficiency by changing the length of the distance between 3'ss and branchpoint. S6
6. Reference S7

Table S1. Oligonucleotide sequences

Name	Sequences	Description
CDC Pre-mRNA	5'-GCATGTAATACGACTCACTATAGGGAGGAAGCTGTCCTTGATG TTGTGGACACTCTGACTGCTGTGCTCCAGGTTGCCACTGGAGTG ATTTCTACCCTCCAGGTAAGGTTTCAGCTTCTCATTATTTAATGTG GTTGGAGGACACATTTTAAGTGTTGTACAGATACAAGAGCTCTAG TCTGGTCCTAACATGAAGACTTGCTCACTCCTACTGCTTGTTATG ACCCCACCACAGGCAGCTCAGATACACTTGGGCAGAAGGGCCT TGGTCCCCTTGATGAAGTCCATTCTTTGAGACCACTAACGTGTCC CCAGGGAGCAGCAAATGATAACCTCTCTCTCTGCCTCTCCTCTTT GCAGGTCAACAAGGAGAACATGGAGAAGGCTCTGACCCCTGAG TTGCTGTCTACTGATCTGGCTCTGCATCC-3'	428nt
CDC mRNA	5'-GCAUGUAAUACGACUCACUAUAGGGAGGAAGCUGUCCUUGA UGUUGUGGACACUCUGACUGCUGUGCUCCAGGUUGCCACUGG AGUGAUUUCUACCCUCCAGGGUCAACAAGGAGAACAUGGAGAA GGCUCUGACCCCUGAGUUGCUGUCUACUGAUCUGGCUCUGCA UCC-3'	171nt
MB-1	5'-/FAM/-CGACGA CTC GAGGTGCATTCA TAT TCGTCG-/DABCYL/-3'	To hybridize with target amplification products for detection
MB-2	5'-/Cy5/-CCACG AGTCAGTGTCCTCAGCGTGG-/DABCYL/-3'	
Padlock probe-1	5'-CCAGTGGCAACCTGGAGCACTTTTTTTCTTGATTACAGTTACGA TTTTTTTGAGTCAGTGTCCTCAGCGTTTTTTCCTGGAGGGTAGAA ATCACT-3'	Padlock probe for free 5'-exon
Padlock probe-2	5'-TCACTCCAGTGGCAATACTGAGATACTGTACGATTTTGAGGTG CATTCATTTTTGCTTCTATTTTCTGGAGCCATCCTGGAGGGTAGAA A-3'	Padlock probe for spliced mRNA
FAM-premier	5'-ATGGCTCCAGAAAATAGAAGCAAAAA-3'	To hybridize with Padlock probe-1 to initiate the RCA reaction
F-primer	5'-AGGAAGCTGTCCTTGATGTT-3'	Forward primer of RT-PCR
R-primer	5'-CTACGTCTCGGTCTAGTCATC-3'	Reverse primer of RT-PCR
5'-ASO	5'- AAA CCT TAC CTG GAG -3'	ASO binding 5' splice site
Mid1-ASO	5'- AGG ACC AGA CTA GAG -3'	ASO binding nt 70 to 84 of intron
Mid2-ASO	5'- AAG GCC CTT CTG CC -3'	ASO binding nt 151 to 165 of intron
BP-ASO	5'- AGA GAG GTT ATC ATT -3'	ASO binding branchpoint sequence
3'-ASO	5'- TTG TTG ACC TGC AAA -3'	ASO binding 3' splice site

Table S2 Inhibitory efficiency by different concentrations of ASO

Name	Concentration (pM)	Percentage of spliced mRNA (%)	Splicing efficiency (%)	Inhibitory efficiency (%)
5'-ASO	0	46.68	100	0
	1	31.61	67.72	32.28
	5	21.27	45.57	54.43
	10	11.00	23.56	76.44
	20	3.45	7.39	92.60
	40	0.10	0.21	99.78
3'-ASO	0	46.68	100	0
	1	36.97	79.20	20.80
	5	29.57	63.35	36.65
	10	16.68	35.73	64.27
	20	04.54	9.73	90.27
	40	03.43	7.35	92.65

Enzyme concentration and incubation time optimization

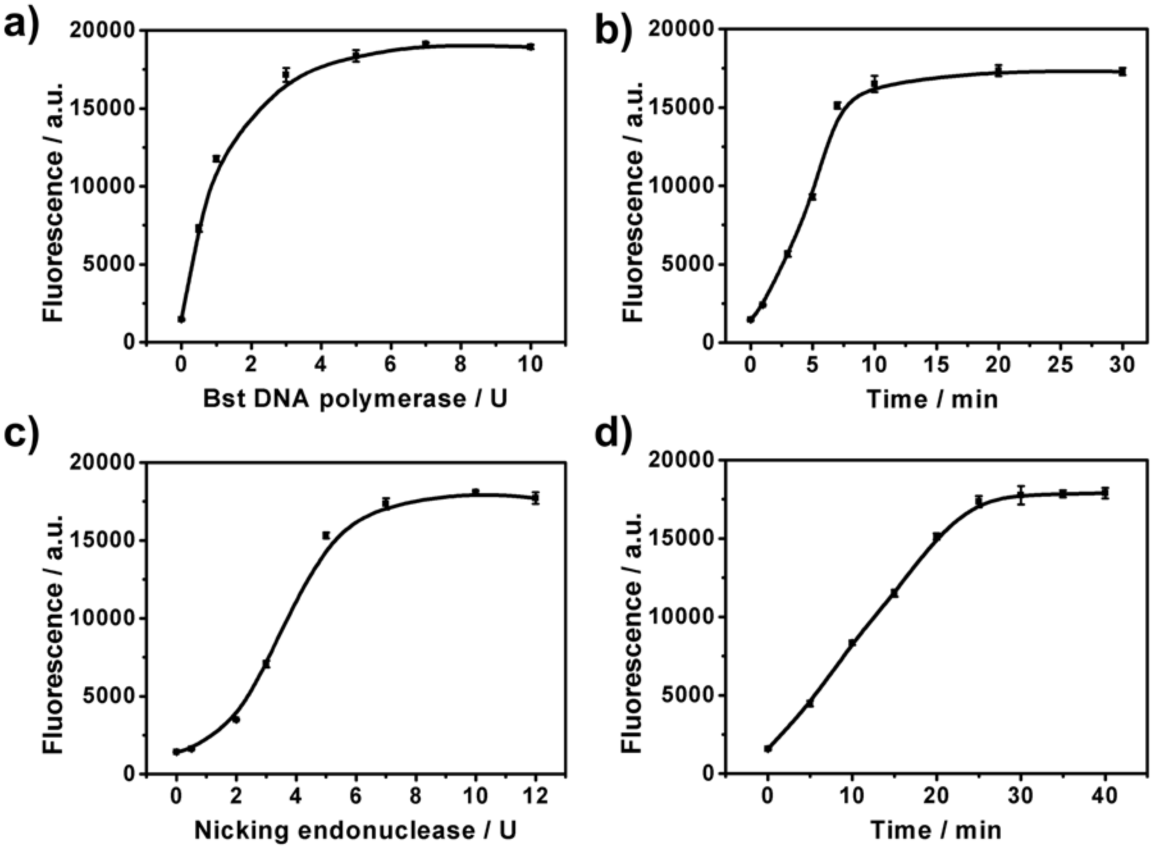

Figure S1. a) Fluorescence signal intensity in the presence of different concentrations of Bst DNA polymerase. **b)** Optimization of the incubation time of Bst DNA polymerase. **c)** Fluorescence signal intensity in the presence of different concentrations of nicking endonuclease (Nb.Mva1269I). **d)** Optimization of the incubation time of Nb.Mva1269I. The assays were carried out in the reaction buffer, containing 10 nM CDC mRNA and 200 nM MB.

Effect of the distance between the branchpoint and the 3' splice site on RNA splicing efficiency

Branchpoint proximity is another important determinant in 3' splice site selection, which affects the second step.[1-3] To further understand the involvement of the distance between the branchpoint and the 3' splice site in each step, we changed the length of the distance in pre-mRNA by shortening the repeat sequence near the branchponit and investigated its effects on RNA splicing efficiency. The distance was shortened by 4, 8, 9, 10 nucleotides (nt) respectively ($CDC_{Δ4}$, $CDC_{Δ8}$, $CDC_{Δ9}$, $CDC_{Δ10}$) (Figure S2 a). When the distance was shortened by 4nt, the amount of free 5'-exon increased while the spliced mRNA decreased compared with that obtained by wild type CDC pre-mRNA. This result indicates that the pre-mRNA underwent the first step efficiently but the second step was blocked, as shown in the Figure S2 b. Distance shortened by 8 nt, the stronger block effect of second-step was presented compared to that of shortened by 4nt. And we observed the almost similar amount of free 5'-exon and spliced mRNA obtained from $CDC_{Δ9}$ and $CDC_{Δ10}$, suggesting that pre-mRNA underwent the first step of splicing efficiently but was completely blocked before the second step when the distance was shortened by 9 nt or more than 9nt. We suggest that the distance was too short to for binding proteins to stabilize 3' splice site binding to the catalytic core, thus the second step of splicing cannot take place.

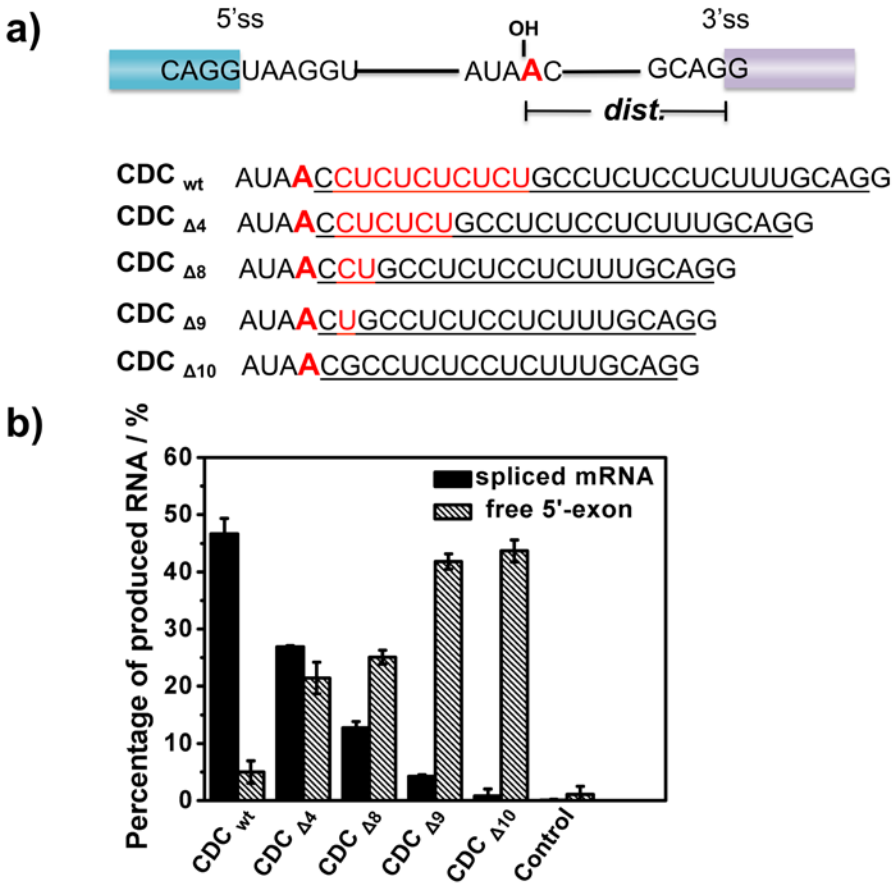

Figure S2. Effect on RNA splicing efficiency by changing the length of the distance between 3'ss and branchpoint. **a)** Schematic illustration of the distance from branchpoint to 3' splice site. The length of the underline sequence is the distance from branchpoint to 3' splice site. CDC $_{wt}$: the wild type of the CDC pre-mRNA; CDC$_{Δ4}$, CDC$_{Δ8}$, CDC$_{Δ9}$, CDC$_{Δ10}$: altered CDC pre-mRNAs which were shortened the CU sequence near the branchponit by 4, 8, 9, 10 nt. **b)** Fluorescence intensity of free 5'-exon and spliced mRNA which were produced by different pre-mRNA (CDC $_{wt}$, CDC$_{Δ4}$, CDC$_{Δ8}$, CDC$_{Δ9}$, CDC$_{Δ10}$), the control indicates the system without pre-mRNA.

Reference

1. A. Cellini, E. Felder and J. J. Rossi, *EMBO. J.*, 1986, **5**, 1023-1030.
2. B. G. Luukkonen and B. Seraphin, *EMBO. J.*, 1997, **16**, 779-792.
3. D. S. Horowitz, *Wiley Interdiscip. Rev. RNA*, 2012, **3**, 331-350.
